# Supplementary material for: Selenium-Fortified Kombucha–Pollen Beverage by In Situ Biosynthesized Selenium Nanoparticles with High Biocompatibility and Antioxidant Activity
Source: Antioxidants (Basel). 2023 Sep 2;12(9):1711. doi: 10.3390/antiox12091711 (PMC10525527; doi:10.3390/antiox12091711)
Supplement: Supplementary file 1 [file antioxidants-12-01711-s001.zip › antioxidants-2549089-supplementary.pdf]

Supplementary Materials

Table S1. Experimental design for optimizing the production of SeNPs from Kombucha fermented pollen.

Run	Factor A (SCOBY)	Factor B (Pollen)	Factor C (Na_2SeO_3)	TPC (µg/mL)	TFC (µg/mL)	HAT (µg/mL)	DPPH (µM)	FRAP (µM)	CUPRAC (µM)	Si (mg/L)	Se^0 (mM)	Se^0 yield (%)
1	15	15	190	8.602	2.056	3.587	436.5	1720	695.7	5	2.043	55.80
2	30	5	190	19.03	4.509	12.84	733.6	4094	1643	4.5	0	0
3	30	15	370	8.671	2.056	3.819	462.1	1754	995.5	5	1.273	17.86
4	30	15	190	13.44	2.294	4.282	490.3	1374	1032	8	1.678	45.84
5	30	5	190	17.27	4.104	16.04	719.8	3779	1313	4	0	0
6	30	15	10	25.54	5.196	19.09	738.4	3726	1723	8	0	0
7	30	15	10	27.55	4.541	25.27	646.7	2764	1531	7	0	0
8	15	5	10	28.33	4.353	19.79	740.3	3414	1477	7	0	0
9	30	25	190	15.18	3.542	8.680	677.5	3044	1140	10	3.456	94.38
10	30	15	190	12.26	2.343	4.861	501.9	1227	1014	5	1.694	46.27
11	30	15	190	8.045	1.769	4.097	514.4	1389	984.3	5	1.980	54.07
12	30	25	190	18.19	3.692	10.67	701.9	3410	1122	13	3.638	99.36
13	15	15	190	9.262	2.468	4.050	569.8	1564	801.8	6	1.940	52.99
14	45	5	370	26.24	4.884	21.41	723.0	648.5	1840	7	0	0
15	15	5	10	28.41	4.272	24.37	654.4	2634	1339	6	0	0
16	45	5	370	26.63	5.065	25.57	719.8	864	1968	5	0	.00
17	30	15	190	11.59	1.875	4.513	642.3	1032	1204	5	2.003	54.72
18	45	25	10	20.19	4.335	12.15	712.1	2633	1248	9	0	0
19	15	25	370	12.24	2.718	4.745	492.9	1314	712.4	9	3.115	43.68
20	30	15	190	12.89	3.136	6.25	682.6	1453	1170	6	2.242	61.22
21	45	15	190	11.08	2.606	4.513	592.3	1573	742.2	8	0	0
22	45	25	10	17.20	3.829	12.15	709.6	2389	1286	9	0	0
23	30	15	370	9.193	2.194	4.166	540.3	1954	969.4	5	1.535	21.53
24	15	25	370	12.89	2.855	4.166	539.1	1139	768.3	6	2.861	40.12
25	45	15	190	12.47	2.824	3.356	600.6	1714	772.0	5	0	0

Table S2. R^2, Adjusted R^2 from the ANOVA analysis.

	R^2	Adjusted R^2
**TPC**	0.971	0.957
**TFC**	0.873	0.83
**HAT**	0.96	0.936
**DPPH**	0.69	0.628
**FRAP**	0.663	0.574
**CUPRAC**	0.952	0.924
**Si**	0.649	0.578
**Se^0**	0.838	0.815
**Se^0 yield**	0.698	0.655

Table S3. Coded equation.

Coded equation
$\mathbf{TPC} = 11.81 + 1.17 \times A - 0.73 \times B - 8.80 \times C - 6.79 \times AB + 5.15 \times AC - 1.56 \times A^2 + 5.50 \times B^2 + 5.81 \times C^2$
$\mathbf{TFC} = 2.53 + 0.40 \times A - 0.51 \times B - 1.37 \times C - 1.21 \times AB + 1.07 \times B^2 + 0.60 \times C^2$
$\mathbf{HAT} = 5.72 + 0.05 \times A - 2.38 \times B - 9.09 \times C - 7.52 \times AB + 4.85 \times AC - 2.21 \times BC - 2.42 \times A^2 + 5.75 \times B^2 + 6.78 \times C^2$
$\mathbf{DPPH} = 570.68 + 52.02 \times A - 38.16 \times B - 60.36 \times C + 106.37 \times B^2$
$\mathbf{FRAP} = 2078.04 - 163.624 \times A - 125.28 \times B - 823.85 \times C - 941.83 \times A^2 + 996.81 \times B^2$
$\mathbf{CUPRAC} = 1050.24 + 4.18 \times A - 173.60 \times B - 322.54 \times C - 314.92 \times AB + 152.77 \times AC - 251.68 \times BC - 277.92 \times A^2 + 274.17 \times B^2 + 274.13 \times C^2$
$\mathbf{Si} = 6.68 + 0.33 \times A + 3.75 \times B - 0.75 \times C + 2.75 \times AC$
$\mathbf{Se^0} = 1.59 + 1.08 \times B + 0.87 \times C + 0.91 \times BC - 0.71 \times C^2$
$\mathbf{Se^0\ yield} = 43.43 + 19.97 \times B + 12.25 \times C + 12.86 \times BC - 31.17 \times C^2$

Table S4. Pearson correlation for the responses of the RSM experimental design.

		TPC	TFC	HAT	DPPH	FRAP	CUPRAC	Si	Se^0	Se^0 yield
**TPC**	Pearson correlation	1	0.916**	0.960**	0.737**	0.407*	0.832**	0.192	-0.568**	-0.512**
	Sig. (2-tailed)		0.000	0.000	0.000	0.044	0.000	0.358	0.003	0.009
	N	25	25	25	25	25	25	25	25	25
**TFC**	Pearson correlation	0.916**	1	0.898**	0.844**	0.517**	0.843**	0.218	-0.566**	-0.516**
	Sig. (2-tailed)	0.000		0.000	0.000	0.008	0.000	0.294	0.003	0.008
	N	25	25	25	25	25	25	25	25	25
**HAT**	Pearson correlation	0.960**	0.898**	1	0.703**	0.380	0.861**	0.067	-0.600**	-0.537**
	Sig. (2-tailed)	0.000	0.000		0.000	0.061	0.000	0.749	0.002	0.006
	N	25	25	25	25	25	25	25	25	25
**DPPH**	Pearson correlation	0.737**	0.844**	0.703**	1	0.546**	0.767**	0.238	-0.466*	-0.343
	Sig. (2-tailed)	0.000	0.000	0.000		0.005	0.000	0.252	0.019	0.093
	N	25	25	25	25	25	25	25	25	25
**FRAP**	Pearson correlation	0.407*	0.517**	0.380	0.546**	1	0.316	0.231	-0.242	-0.164
	Sig. (2-tailed)	0.044	0.008	0.061	0.005		0.124	0.266	0.243	0.433
	N	25	25	25	25	25	25	25	25	25
**CUPRAC**	Pearson correlation	0.832**	0.843**	0.861**	0.767**	0.316	1	-0.031	-0.557**	-0.458*
	Sig. (2-tailed)	0.000	0.000	0.000	0.000	0.124		0.885	0.004	0.021
	N	25	25	25	25	25	25	25	25	25
**Si**	Pearson correlation	0.192	0.218	0.067	0.238	0.231	-0.031	1	0.325	0.353
	Sig. (2-tailed)	0.358	0.294	0.749	0.252	0.266	0.885		0.112	0.083
	N	25	25	25	25	25	25	25	25	25
**Se^0**	Pearson correlation	-0.568**	-0.566**	-0.600**	-0.466*	-0.242	-0.557**	0.325	1	0.942**
	Sig. (2-tailed)	0.003	0.003	0.002	0.019	0.243	0.004	0.112		0.000
	N	25	25	25	25	25	25	25	25	25
**Se^0 yield**	Pearson correlation	-0.512**	-0.516**	-0.537**	-0.343	-0.164	-0.458*	0.353	0.942**	1
	Sig. (2-tailed)	0.009	0.008	0.006	0.093	0.433	0.021	0.083	0.000	
	N	25	25	25	25	25	25	25	25	25

**. Correlation is significant at the 0.01 level (2-tailed); *. Correlation is significant at the 0.05 level (2-tailed).

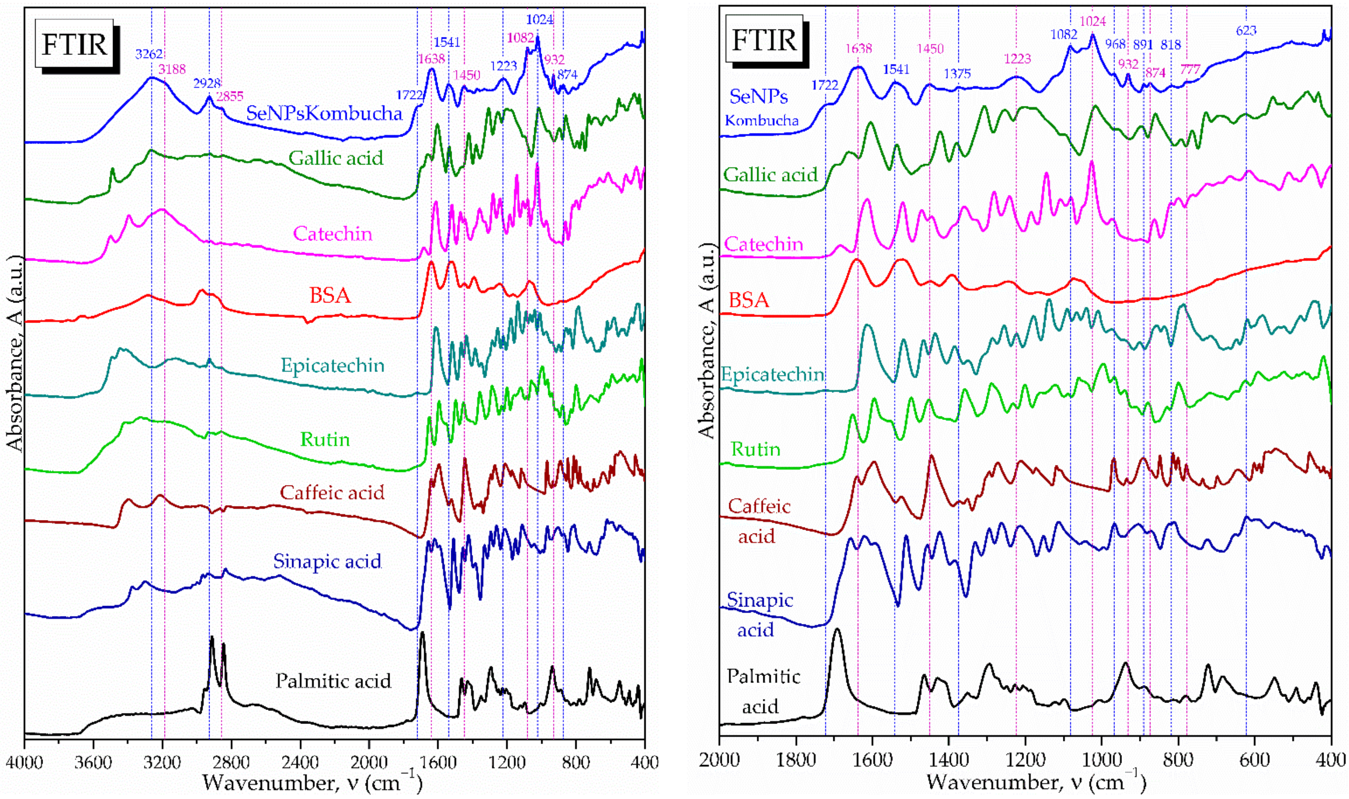

(a) (b)

Figure S1. FTIR analysis of SeNPs Kombucha in comparison with different compounds: (**a**) Full spectral range; (**b**) Fingerprint region.

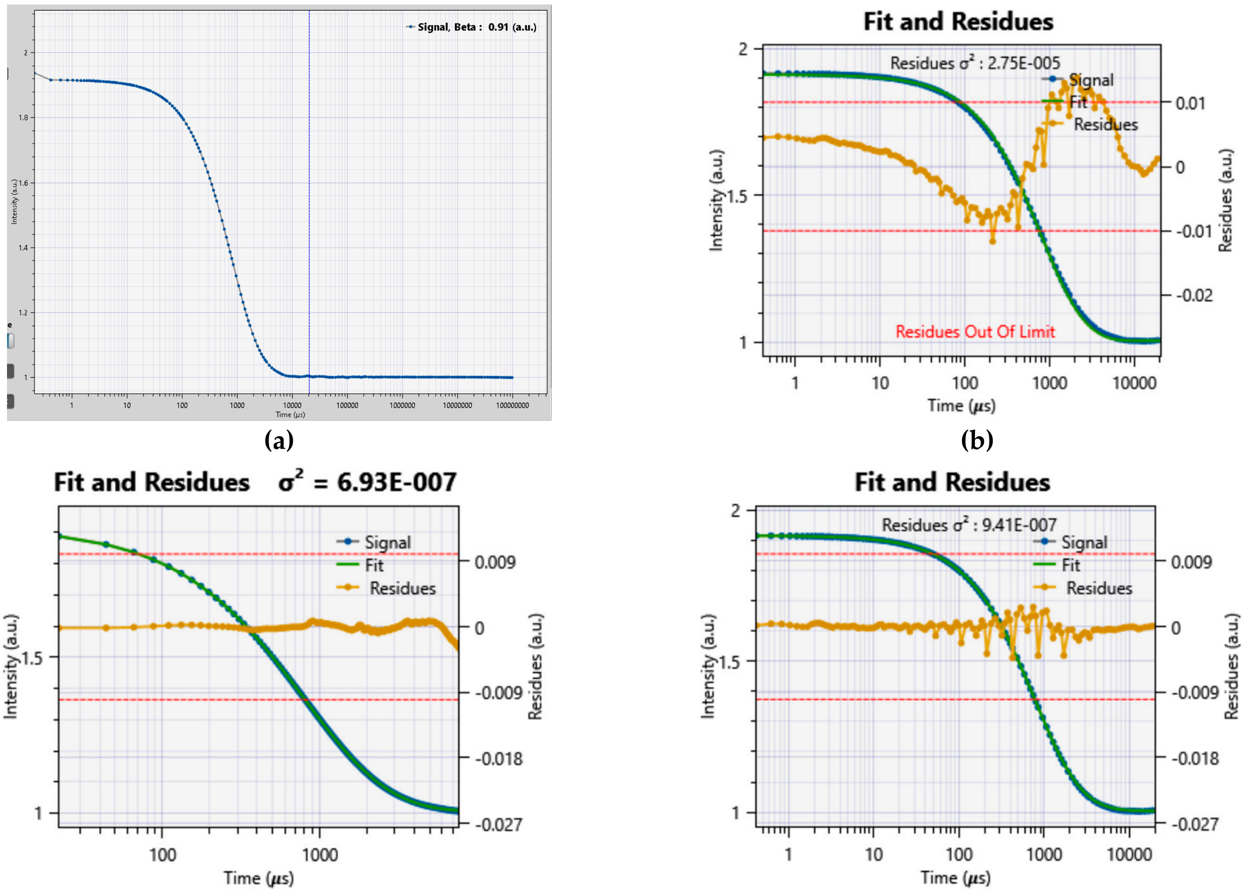

(a) (b) (c) (d)

Figure S2. DLS analysis: (**a**) autocorrelation function for SeNPs Kombucha; (**b**) simulation of autocorrelation function for Cumulants method; (**c**) simulation of autocorrelation function for Pade Laplace (PL) method; (**d**) simulation of autocorrelation function for SBL method.

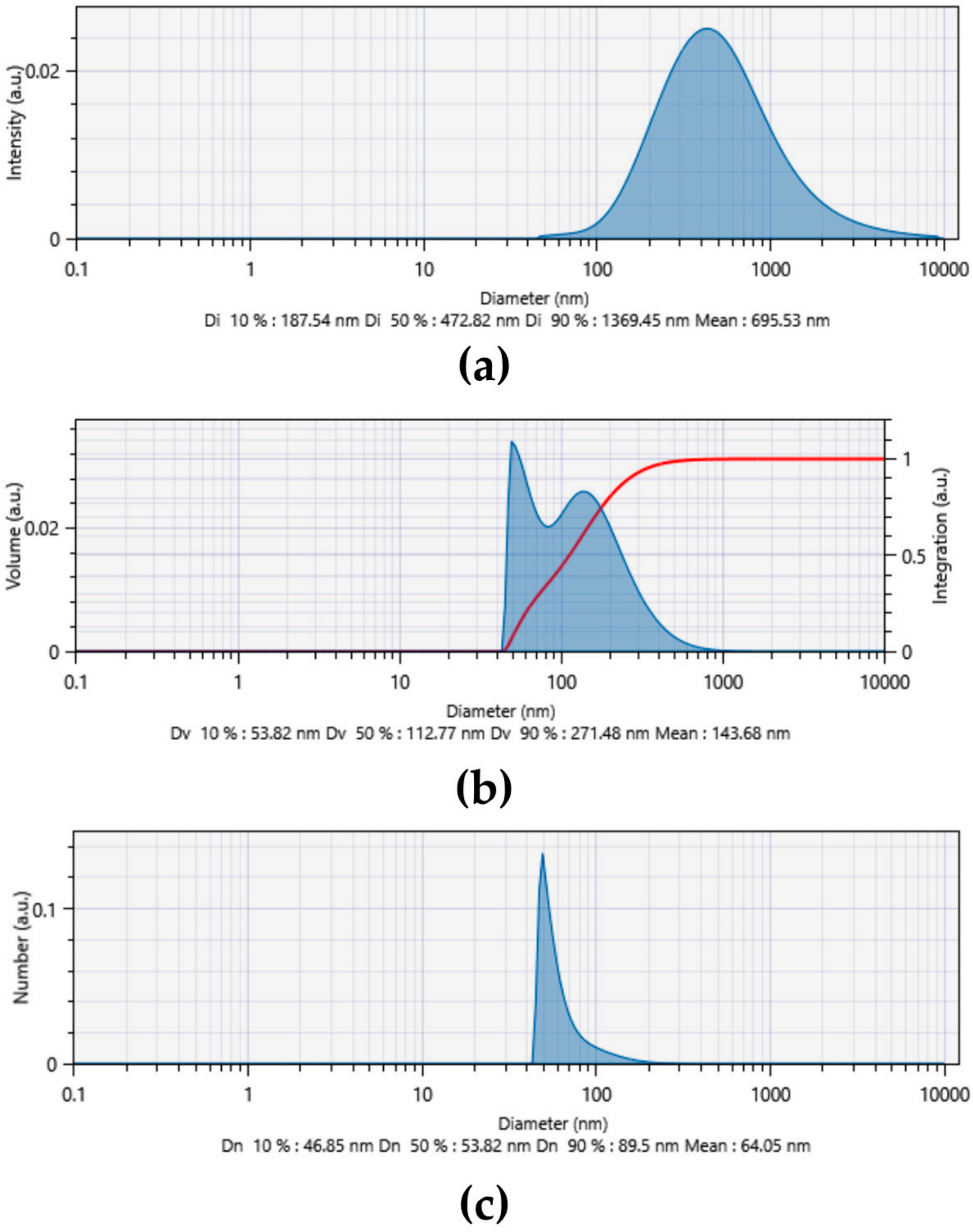

Figure S3. DLS analysis of SeNPs: (**a**) Intensity; (**b**) Volume; (**c**) Number.

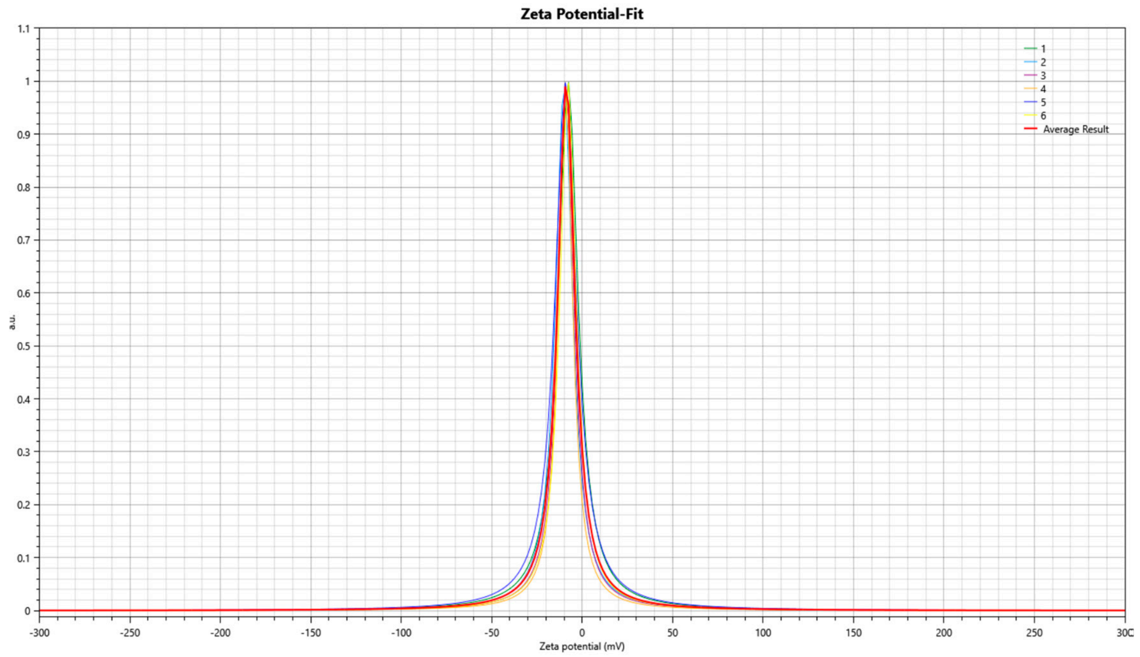

Figure S4. Zeta potential analysis of SeNPs.

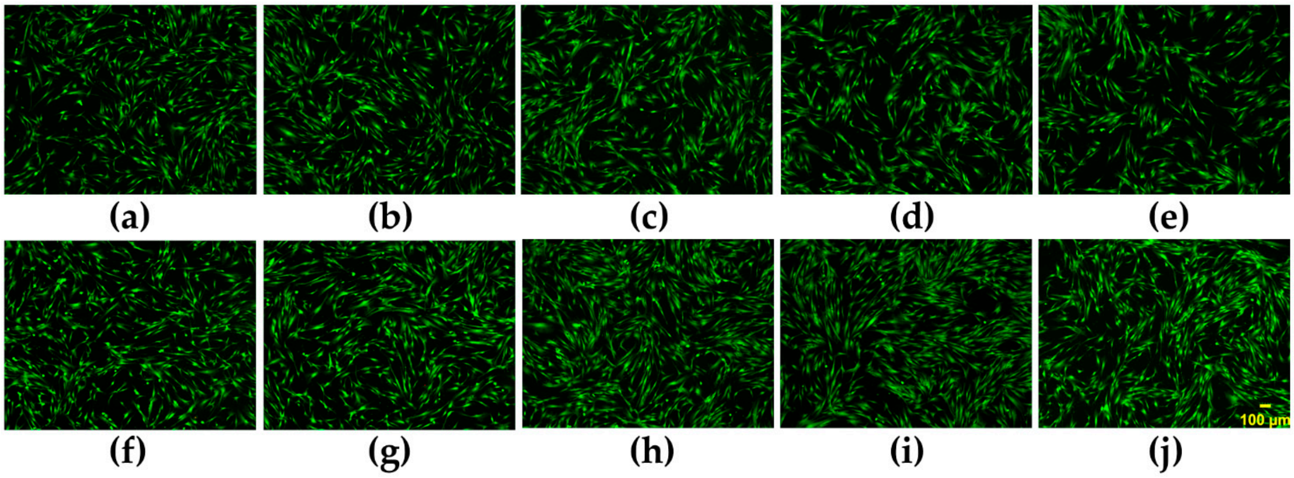

Figure S5. Biocompatibility of Kombucha beverage: (**a**–**j**) LIVE/DEAD assay (live cells – green fluorescence, dead cells – red fluorescence): (**a**) 0.1 mg/mL KPol5; (**b**) 1 mg/mL KPol5; (**c**) 3 mg/mL KPol5; (**d**) 5 mg/mL KPol5; (**e**) 7 mg/mL KPol5; (**f**) 0.1 mg/mLKPol15; (**g**) 1 mg/mL KPol15; (**h**) 3 mg/mL KPol15; (**i**) 5 mg/mL KPol15; (**j**) 7 mg/mL KPol15; KPol5 – Kombucha beverage with 30 mL SCOBY, 5 g pollen, and 190 mg sodium selenite; KPol15 – Kombucha beverage with 30 mL SCOBY, 15 g pollen, and 190 mg sodium selenite.

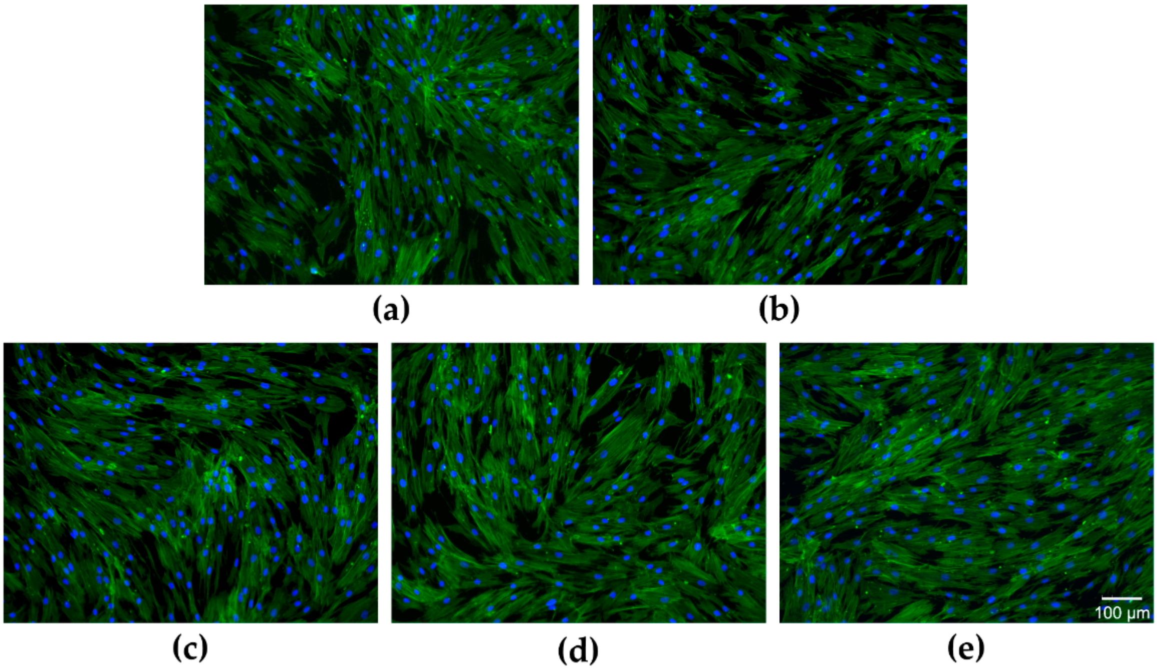

Figure S6. Cell morphology – Fluorescence microscopy images after labelling the actin cytoskeleton with Alexa Fluor 488-coupled phalloidin (green fluorescence), and the nuclei with DAPI (blue fluorescence): (**a**) Untreated cells (C-; Negative cytotoxicity control); HGF-1 cells treated with: (**b**) 3 mg/mL K; (**c**) 3 mg/mL KPol5; (**d**) 5 mg/mL KPol15; (**e**) 7 mg/mL KPol25; K – Kombucha beverage with 30 mL SCOBY; KPol5 – Kombucha beverage with 30 mL SCOBY, 5 g pollen, and 190 mg sodium selenite; KPol15 – Kombucha beverage with 30 mL SCOBY, 15 g pollen, and 190 mg sodium selenite, KPol25 – Kombucha beverage with 30 mL SCOBY, 25 g pollen, and 190 mg sodium selenite.

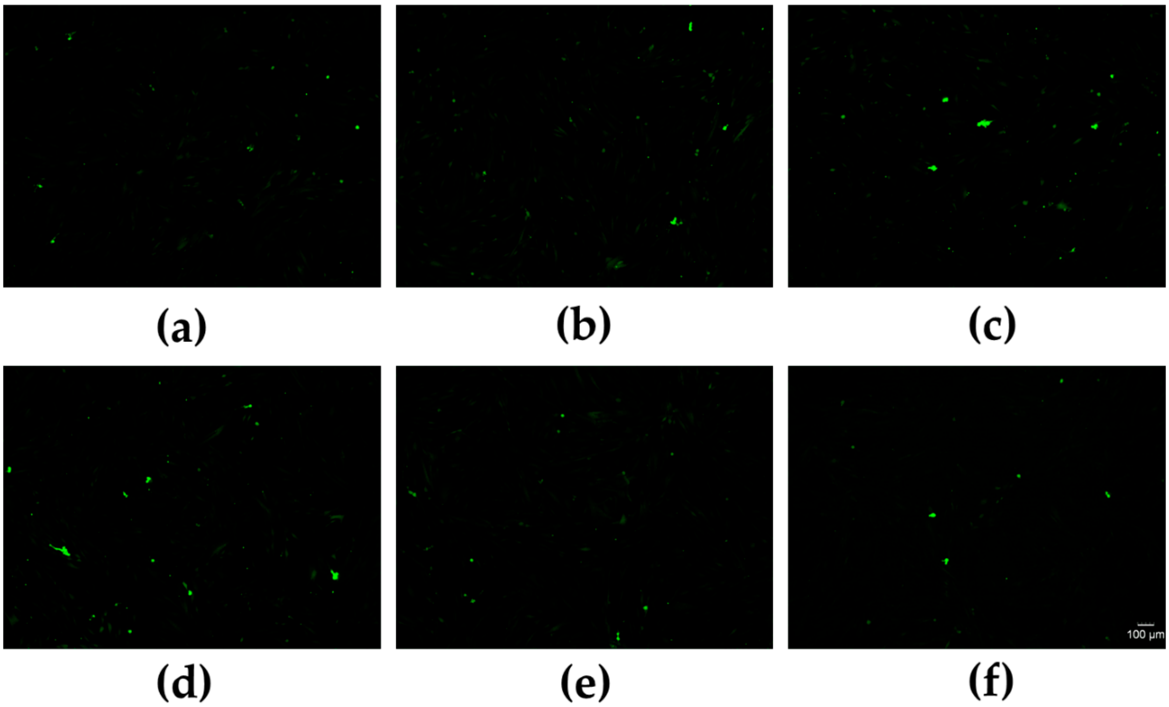

Figure S7. In vitro antioxidant activity of Kombucha beverage: (**a-f**) Fluorescence microscopy images after labelling total intracellular ROS with H_2DCFDA (green fluorescence): (**a**) 3 mg/mL KPol5; (**b**) 5 mg/mL KPol5; (**c**) 7 mg/mL; (**d**) 3 mg/mL KPol15; (**e**) 5 mg/mL KPol15; (**f**) 7 mg/mL KPol15; KPol5 – Kombucha beverage with 30 mL SCOBY, 5 g pollen, and 190 mg sodium selenite; KPol15 – Kombucha beverage with 30 mL SCOBY, 15 g pollen, and 190 mg sodium selenite.

Table S5. EDX analysis of fresh polyfloral pollen (P).

Element	Weight %	σ
**C**	62.8	0.8
**O**	35.6	0.8
**Al**	0.8	0.1
**K**	0.4	0.1
**P**	0.2	0.1

Table S6. EDX analysis of PK.

Element	Weight %	σ
**C**	64.8	0.7
**O**	32.6	0.6
**N**	2.6	0.8

Table S7. EDX analysis of PK25.

Element	Weight %	σ
**C**	67.0	0.5
**O**	30.6	0.4
**Se**	2.5	0.3

Table S8. EDX analysis of BCK.

Element	Weight %	σ
**C**	56.7	0.3
**O**	41.8	0.3
**Al**	0.6	0.0
**P**	0.5	0.0
**S**	0.2	0.0
**Ca**	0.2	0.0
**K**	0.1	0.0

Table S9. EDX analysis of BCKPol5.

Element	Weight %	σ
**O**	54.2	0.4
**C**	45.3	0.4
**Al**	0.4	0.0

Table S10. EDX analysis of BCKPol15.

Element	Weight %	σ
**C**	52.4	0.5
**O**	45.9	0.5
**P**	0.5	0.0
**Al**	0.4	0.0
**K**	0.3	0.0
**S**	0.2	0.0
**Ca**	0.2	0.0
**Na**	0.2	0.0

Table S11. EDX analysis of BCKPol25.

Element	Weight %	σ
**C**	51.2	0.4
**O**	46.7	0.4
**K**	0.6	0.0
**Al**	0.6	0.0
**P**	0.4	0.0
**Ca**	0.2	0.0
**Se**	0.2	0.1
**S**	0.2	0.0

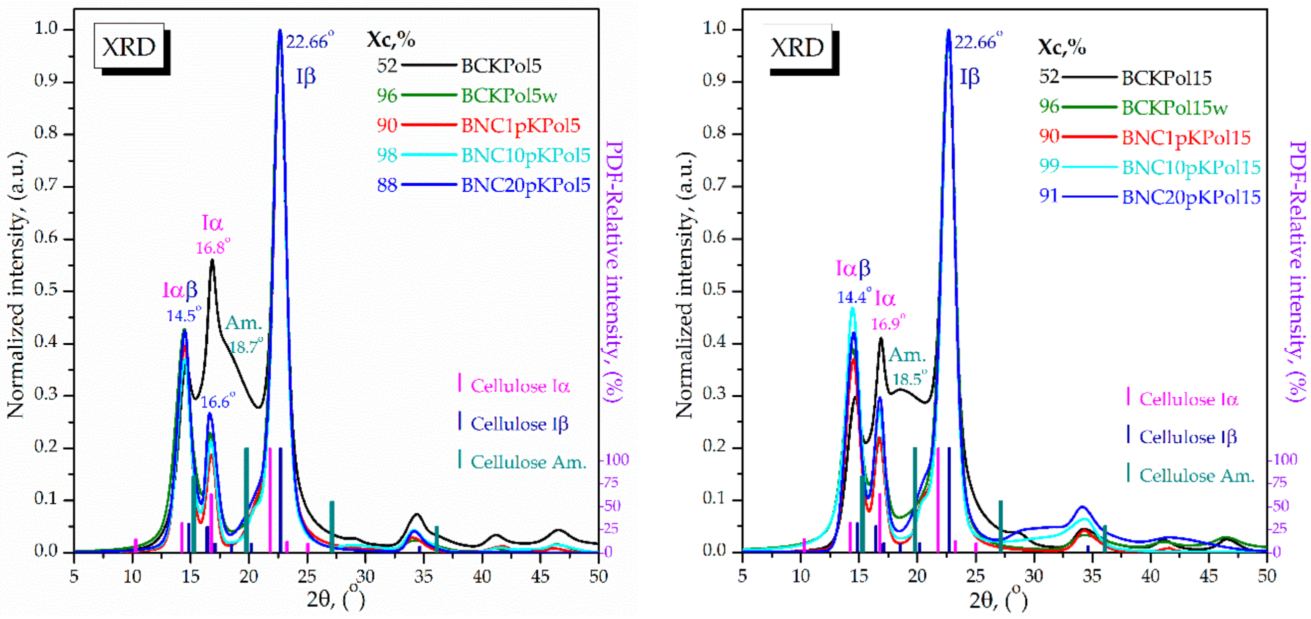

(a) **(b)**

Figure S8. XRD analyses of bacterial celluloses membranes (BC) obtained by purification and microfluidization: (**a**) BCKPol5 – Bacterial cellulose from Kombucha beverage prepared with 30 mL SCOBY, 5 g pollen, and 190 mg sodium selenite; BCKPol5w – Purified/washed bacterial cellulose; BNC1pKPol5 – Bacterial nanocellulose after 1 pass of microfluidization; BNC10pKPol5 – Bacterial nanocellulose after 10 passes of microfluidization; BNC20pKPol5 – Bacterial nanocellulose after 20 passes of microfluidization; (**b**) BCKPol15 – Bacterial cellulose from Kombucha beverage prepared with 30 mL SCOBY, 15 g pollen, and 190 mg of sodium selenite; BCKPol15w – Purified/washed bacterial cellulose; BNC1pKPol15 – Bacterial nanocellulose after 1 pass of microfluidization; BNC10pKPol15 – Bacterial nanocellulose after 10 passes of microfluidization; BNC20pKPol15 – Bacterial nanocellulose after 20 passes of microfluidization. The purification of BCKPol5 and BCKPol15 increases the crystallinity degree from 52% to 96% in washed membranes (w), while 10 passes of microfluidization induce a partial arrangement of amorphous cellulose into a Iαβ structure, as suggested by the increased intensity of the Iαβ peak around 14.5° and increased crystallinity up to 98-99%. Subsequent microfluidization up to 20 passes shortens the cellulose microfibrils and partially disintegrate the Iα and Iαβ structure into amorphous cellulose, decreasing the crystallinity degree to 88% for BNC20pKPol5, respectively 91% for BNC20pKPol15.

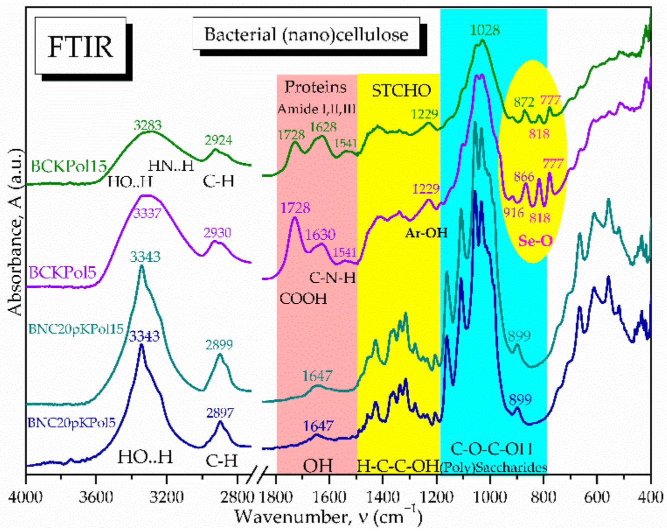

Figure S9. ATR-FTIR spectra of initial membranes of bacterial celluloses (BC) compared with the corresponding bacterial nanocelluloses (BNC) obtained by purification and microfluidization; BCKPol5 – Bacterial cellulose from Kombucha beverage prepared with 30 mL SCOBY, 5 g pollen, and 190 mg sodium selenite; BNC20pKPol5 – Bacterial nanocellulose after 20 passes of microfluidization; BCKPol15 – Bacterial cellulose from Kombucha beverage prepared with 30 mL SCOBY, 15 g pollen, and 190 mg of sodium selenite; BNC20pKPol15 – Bacterial nanocellulose after 20 passes of microfluidization.

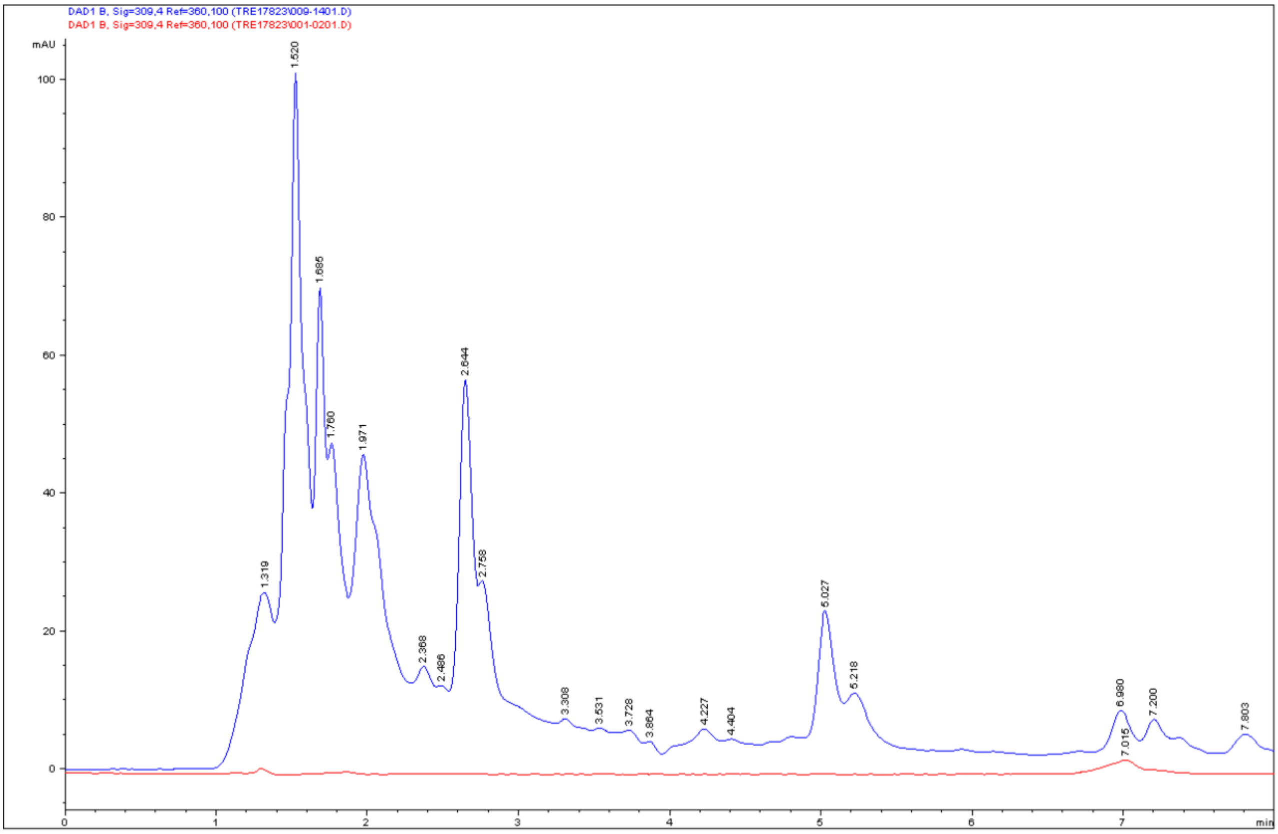

Figure S10. HPLC-DAD chromatogram analysis of black tea (blue) and t-resveratrol reference material (red); t-resveratrol (tr = 7.015 min for reference material, tr = 6.980 min. for sample; λ= 309 nm)

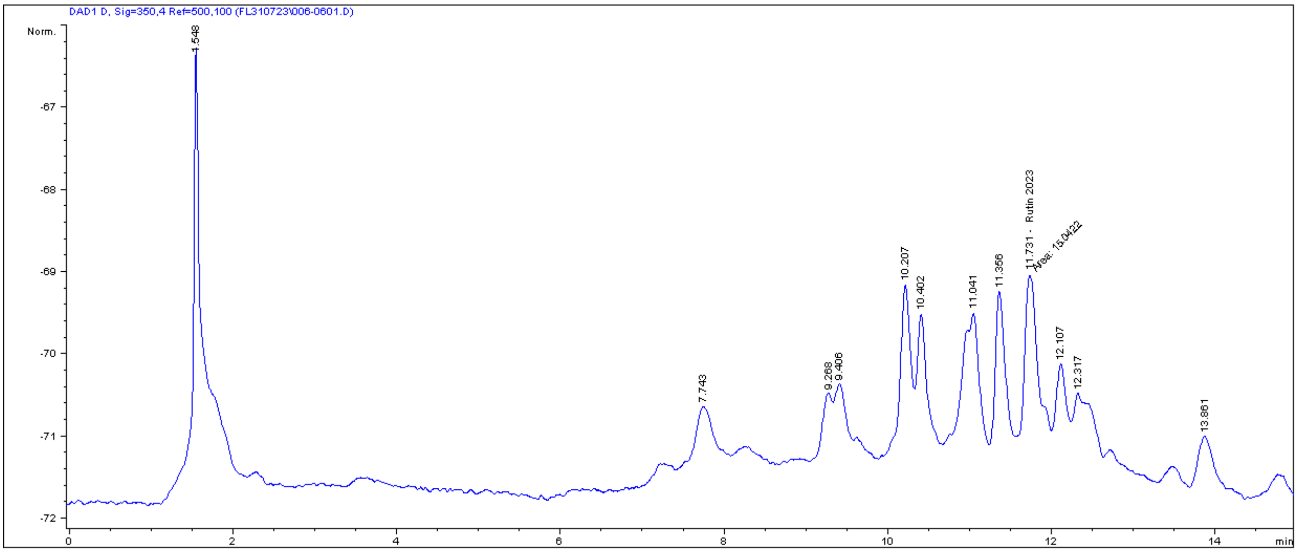

Figure 11. HPLC-DAD chromatogram analysis of black tea sample: quercetin-3-rutinoside (tr = 11.73 min, λ= 350 nm)
